# Digital health information on autoinflammatory diseases: a YouTube quality analysis

**DOI:** 10.1007/s00296-022-05243-9

**Published:** 2022-11-14

**Authors:** Mareen Sasse, Sarah Ohrndorf, Andriko Palmowski, Annette D. Wagner, Gerd Rüdiger Burmester, Anne Pankow, Martin Krusche

**Affiliations:** 1 Department of Rheumatology and Clinical Immunology, Charité-Universitätsmedizin Berlin, Berlin, Germany; 2 Hannover Medical School, Department of Nephrology, Hannover, Germany; 3 Center for Internal Medicine and Rheumatology, Universitätsklinikum Hamburg-Eppendorf, Hamburg, Germany; 4 Section for Biostatistics and Evidence-Based Research, the Parker Institute, Bispebjerg and Frederiksberg Hospital, Copenhagen, Denmark

**Keywords:** Periodic fever syndromes, Familial Mediterranean fever, AOSD, Social media, Video analysis

## Abstract

Getting access to specialists for autoinflammatory diseases (AID) can be challenging. Therefore, an increasing number of patients and healthcare professionals are seeking information on AID via the Internet, using the video platform YouTube, for example. However, the quality of such videos has not yet been evaluated. A YouTube search was conducted to assess videos about AID to evaluate the quality and usefulness from both the patient’s and healthcare professional´s perspectives. Video duration, number of views, likes, dislikes, comments, and uploading source on various AID were extracted. Video quality was evaluated by the modified global quality scale (GQS). The reliability was assessed by the modified five-point DISCERN score. In total, 140 videos were screened of which 105 videos met the inclusion criteria for further analysis. Based on the GQS, the overall quality of videos for patients was found to be low in 64.8%, intermediate in 27.6%, and high in 7.6% of videos. The quality of videos for professionals was similar (54.3% low, 23.8% intermediate, and 21.9% of high quality). Videos were more often targeting medical professionals (65.7%) and less often patients (34.3%). This analysis demonstrates that the majority of videos regarding AIDs are of limited quality. Available videos more often address users with a professional medical background. Only a small proportion of existing videos provide understandable and useful information for AID patients. Thus, there is a strong need to develop high-quality and audience-oriented videos in the context of educational campaigns for these rare disease groups.

**Supplementary Information:**

The online version contains supplementary material available at 10.1007/s00296-022-05243-9.

## Background

Autoinflammatory diseases (AID) refer to a family of disorders caused by inappropriate activation of inflammatory mechanisms [1]. Unlike autoimmune diseases, several genetic pathways of the innate immune system lead to inflammatory responses [2]. Commonly, AID present with periodic fever episodes, often combined with other clinical symptoms, such as arthralgia, skin rashes, or peritonitis. Given the rare disease character and the variety of clinical symptoms, finding the right diagnosis is often challenging for physicians [3] due to a lack of awareness of this rare disease group. Linked to this, patients with AID are often exposed to a long journey until they receive adequate diagnosis and treatment [4]. Furthermore, specialists on AID are scarce and often have limited time for patient education [5]. Therefore, finding and getting access to reliable information concerning AID can often be challenging for affected patients and their relatives [4]. These obstacles have an impact on the emergence of further disease-related issues, such as depression, loss of workplace, and social deprivation [6].

Notably, the influence of social media in healthcare has grown rapidly in recent years [7]. Several studies demonstrate that digital platforms have become a popular source of health information [7], not only for patients and relatives but also as a considerable tool for professionals to seek and exchange information [8]. YouTube is currently the most often used video platform [9] and has become an integral part of the everyday life for many young people in particular [10]. However, as a consumer-generated platform with no upload filter on a video´s credibility, there is a potential for misleading content [11]. Given the already limited access to information concerning AID, little is known about the quality and reliability of uploaded videos on this topic so far.

This study aims at evaluating the quality of available videos related to AID on YouTube. Furthermore, an additional analysis on the two target groups—physicians and patients—has been carried out to assess the usefulness of these videos as a source of information from these two perspectives.

## Materials and methods

A YouTube search was conducted in January 2022. Selected keywords were “autoinflammatory diseases” (AID), “periodic fever syndrome” (PFS), “familial Mediterranean fever” (FMF), “Cryopyrin-associated periodic syndrome” (CAPS), “tumor necrosis factor receptor-associated periodic syndrome” (TRAPS), “adult-onset of Still´s disease” (AOSD), and “systemic juvenile idiopathic arthritis” (SJIA). The default setting ‘relevance’ was used as a filter, as it is the most common YouTube user setting and had also been applied in previous studies [12].

For every keyword, the top 20 videos in order of relevance were included. Only videos in English were included in the analysis. Exclusion criteria were duplicated videos, videos other than English and those with irrelevant content.

Assessment for eligibility was performed by two independent reviewers (MS and AP), following inclusion and exclusion criteria. In case of discrepancies in the evaluation of eligibility by the two reviewers, a third reviewer acted as an arbiter (MK).

Video duration, number of views, likes, dislikes, comments, uploading source, and target audience were extracted. We subsumed medical doctors of any speciality as “professionals” and patients and relatives as “private persons” The uploading source was categorized into professionals, private persons, pharmaceutical industry, and others according to the indication of individual video. If a source could not be attributed, it was classified as ‘other’. The target audience was categorized by the reviewers into patients/relatives and professionals. This was done according to the type of language (i.e., use of medical terminology), the setting (i.e., congress recording), and the general medical expertise required to understand the videos.

The quality of the video’s information was evaluated based on the modified global quality scale (GQS) [13] used in several studies concerning quality evaluation in YouTube videos [12, 14, 15].

The GQS includes five rating scores; from one (poor quality, not at all useful for patients) to five (excellent quality, very useful for patients). A higher rating score of the GQS indicates a better video quality.

The questionnaire was originally designed to represent the quality of videos for patients, but not for physicians. Nevertheless, a video can be of good quality for professionals but of no or limited value for patients and vice versa, e.g., due to the frequent use of medical terminology. Therefore, depending on the target group, two different forms of the GQS were used and the GQS was split into one version for patients (GQSpat) and one version for professionals (GQSprof), respectively (Table 1A). In line with previous studies, the GQS was divided into three categories: low quality (score 1–2), intermediate quality (score 3), and high quality (score 4–5) [12, 16].

**Table 1:** **A** Global quality scale (GQS) for patients (GQSpat) and professionals (GQSprof) **B** DISCERN tool

A	
Global quality scale patients (GQSpat)	Global quality scale professionals (GQSprof)
Low quality	
1. Poor quality, poor flow of the video, most information missing, not at all useful for patients	1. Poor quality, poor flow of the video, most information missing, not at all useful for professionals
2. Generally poor quality and poor flow, some information listed but many important topics missing, of very limited use to patients	2. Generally poor quality and poor flow, some information listed but many important topics missing, of very limited use to professionals
Intermediate quality	
3. Moderate quality, suboptimal flow, some important information is adequately discussed but others poorly discussed, somewhat useful for patients	3. Moderate quality, suboptimal flow, some important information is adequately discussed but others poorly discussed, somewhat useful for professionals
High quality	
4. Good quality and generally good low. Most of the relevant information is listed, but some topics not covered, useful for patients	4. Good quality and generally good low. Most of the relevant information is listed, but some topics not covered, useful for professionals
5. Excellent quality and flow, very useful for patients	5. Excellent quality and flow, very useful for professionals

B
DISCERN tool
1. Is the video clear, concise, and understandable?
2. Are valid sources cited? (from valid studies, physiatrists, or rheumatologists)
3. Is the information provided balanced and unbiased?
4. Are additional sources of information listed for patient reference?
5. Does the video address areas of controversy/uncertainty?

The reliability of the videos was assessed by the modified five-point DISCERN score, which has also been used by previous studies [13]. The questionnaire is scored from one to five, based on five questions. Higher scores represent greater reliability (Table 1B).

To assess view and like ratios, we used the Video Power Index (VPI; like ratio*view ratio/100) [17]. The view ratio was calculated by the number of views/days and the like ratio by like*100/ (like + dislike).

For statistical analyses, SPSS version 28.0.1 was used. All data were tested for normality with the Kolmogorov–Smirnov test. Nominal variables were compared by Chi-square test. Variables that were non-normally distributed were shown with a median (minimum–maximum). To assess the differences between reliability (DISCERN) and quality (GQSpat/GQSprof) scores, the Mann–Whitney *U* test and the Kruskal–Wallis test were used. Correlations were assessed with the Pearson correlation coefficient. The level of statistical significance α was set at 0.05.

## Results

In total, 140 videos were screened. Five videos were excluded due to language other than English, seven were found to be duplicates, and 23 were found not to be suitable (e.g., they did not address the medical topic). A total of 105 videos were further analyzed (see Figure SI 1 in the Supplementary Information). Cohen’s kappa statistic was 0.807 (*p* < 0.001). The median video length was 6.75 min and the median number of views was 1.569. Videos received a median of 15 likes and 0 dislikes. There was a significant negative correlation between the length of the video and the number of clicks (*r* = − 0.196, *p* < 0.05). Video characteristics stratified by disease group are presented in Table 2.

**Table 2:** Basic characteristics—presented by the medians and ranges (minimum–maximum)

	Median (range)							
	Total included (*n* = 105)	AID (*n* = 19)	PFS (*n* = 15)	FMF (*n* = 16)	CAPS (*n* = 13)	TRAPS (*n* = 3)	AOSD (*n* = 20)	SJIA (*n* = 19)
Duration in minutes	6.75 (0.58–86.33)	16.13 (0.63–75.18)	3.78 (1.18–54.02)	10.80 (1.58–51.35)	3.48 (0.58–23.27)	6.10 (3.03–86.33)	9.88 (1.47–48.28)	7.28 (1.65–59.67)
Total views	1569 (24–102.928)	1672 (223–32.930)	2560 (24–36.732)	2182.50 (40–24.842)	635 (26–32.932)	1392 (520–2963)	1029 (192–20.011)	2317 (202–102.928)
No. of likes	15 (0–1275)	18 (0–357)	26 (0–271)	22 (0–323)	8 (0–357)	11 (8–14)	20.50 (3–133)	15 (0–1275)
No. of dislikes	0 (0–17)	0 (0–7)	0 (0–0)	0 (0–17)	0 (0–7)	0 (0–0)	0 (0–0)	0 (0–0)
GQS (patients)	2 (1–5)	3 (1–4)	3 (1–4)	2 (1–5)	2 (1–4)	2 (1–2)	1.50 (1–4)	2 (1–3)
GQS (professionals)	2 (1–5)	3 (1–5)	1 (1–5)	2 (1–5)	1 (1–5)	2 (1–4)	2 (1–4)	3 (1–5)
DISCERN	2 (0–5)	3 (0–5)	2 (1–4)	2 (0–4)	1 (0–4)	1 (1–4)	2 (0–3)	2 (0–4)
VPI *n* = 96	1.43 (0–48.78)	1.59 (0.21–16.11)	1.58 (0.27–10.97)	2 (0.46–10.46)	0.72 (0–16.11)	0.82 (0.66–1.22)	1.82 (0.19–27.25)	1.16 (0.11–48.78)

*AID* autoinflammatory diseases, *PFS* periodic fever syndrome, *FMF* familial Mediterranean fever, *CAPS* cryopyrin-associated periodic syndrome, *TRAPS* tumor necrosis factor receptor-associated periodic syndrome, *AOSD* adult onset of Still ´s disease, *SJIA* systemic juvenile idiopathic arthritis, *GQS* global quality scale, 1 (low quality)—5 (high quality); *DISCERN* reliability of the videos 0 (low reliability)—5 (high reliability)

### Target group and uploading sources

**Fig. 1:**
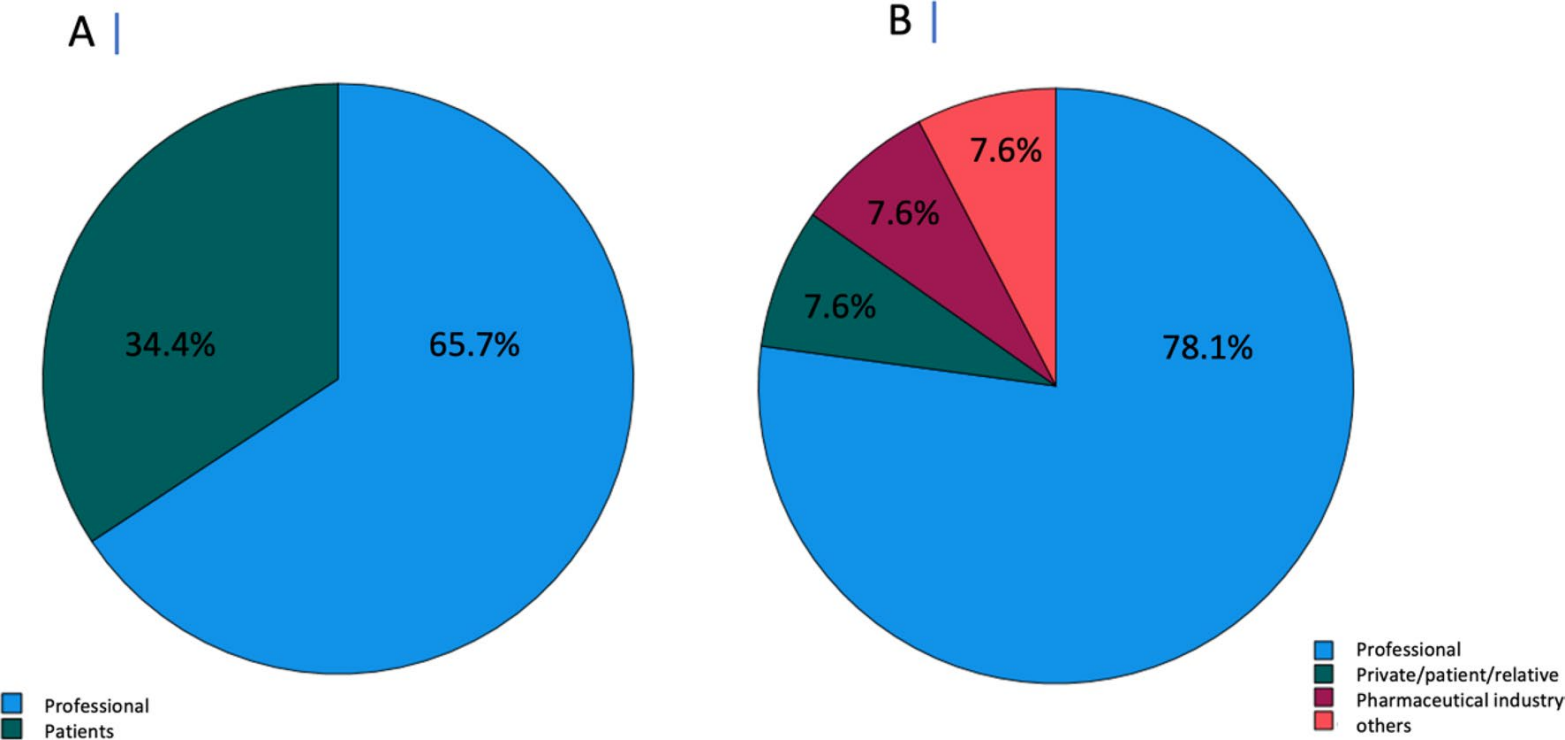
**A **Videos targeted at professionals (blue) and private persons, patients and relatives (green). **B **Videos created by professionals (blue), private persons, patients and relatives (green), pharmaceutical industry (red), and others (orange) in percentage

Most videos were uploaded to inform healthcare professionals about the pathophysiology, symptoms, and therapies of AID (*n* = 69, 65.7%). Only a small proportion targeted patients or their relatives (*n* = 36, 34.4%) (Fig. 1A). The most frequently uploaded videos were created by healthcare professionals (*n* = 82, 78.1%), followed in equal parts by private persons, patients and relatives, the pharmaceutical industry, and others (each *n* = 8, 7.6%) (Fig. 1B).

### Quality of content

**Fig. 2:**
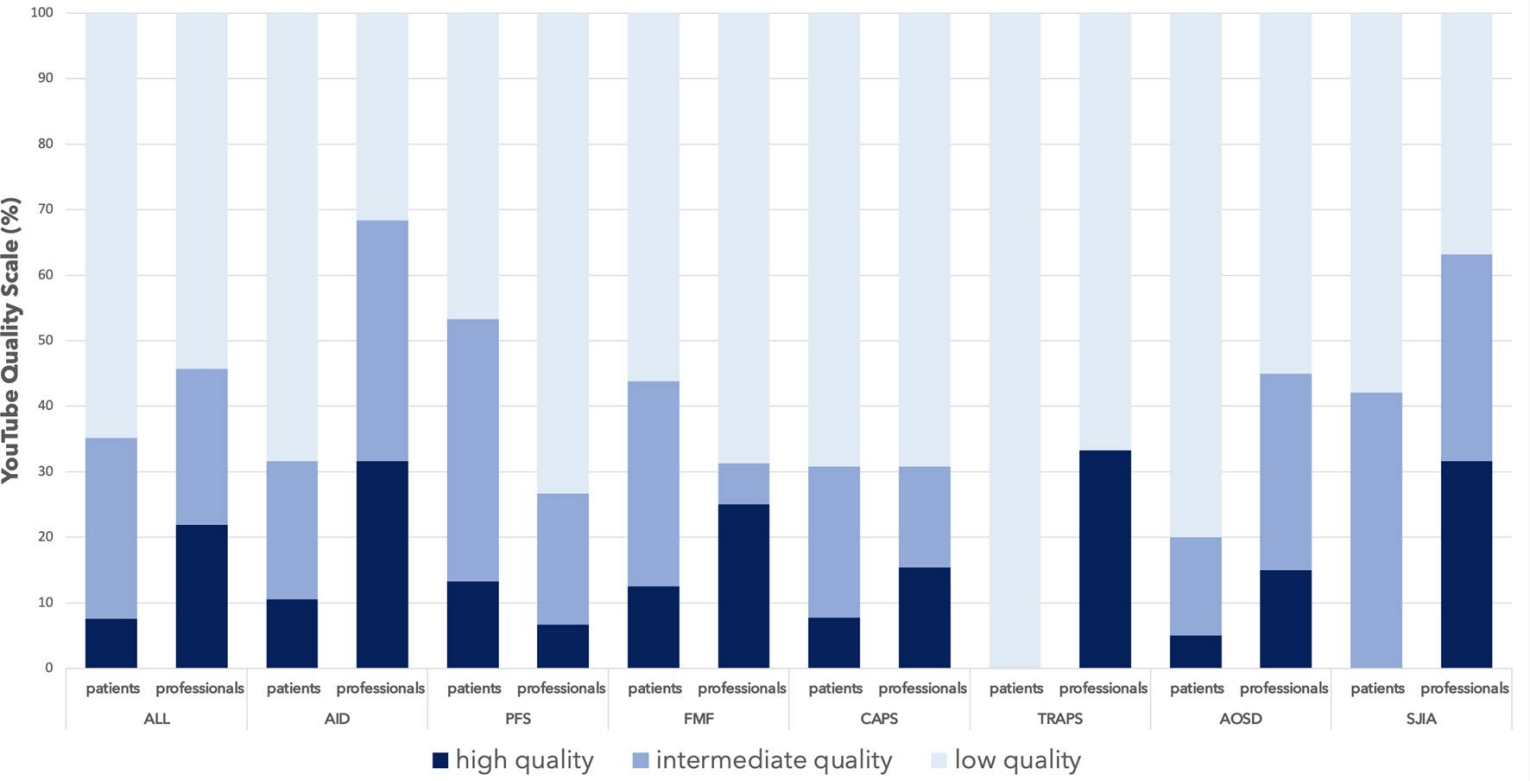
Quality Scale in percentage for overall videos, ALL; *AID* autoinflammatory diseases, *PFS* periodic fever syndrome, *FMF* familial Mediterranean fever, *CAPS* cryopyrin-associated periodic syndrome, *TRAPS* tumor necrosis factor receptor-associated periodic syndrome, *AOSD* adult onset of Still ´s disease, *SJIA* systemic juvenile idiopathic arthritis

Based on the GQS, the overall quality of videos for patients was found to be high in 7.6%, intermediate in 27.6%, and low in 64.8%. The quality of videos for professionals showed a similar pattern: 22.9% were found to be of high, 22.9% of intermediate, and 54.3% of low quality (Fig. 2).

**Fig. 3:**
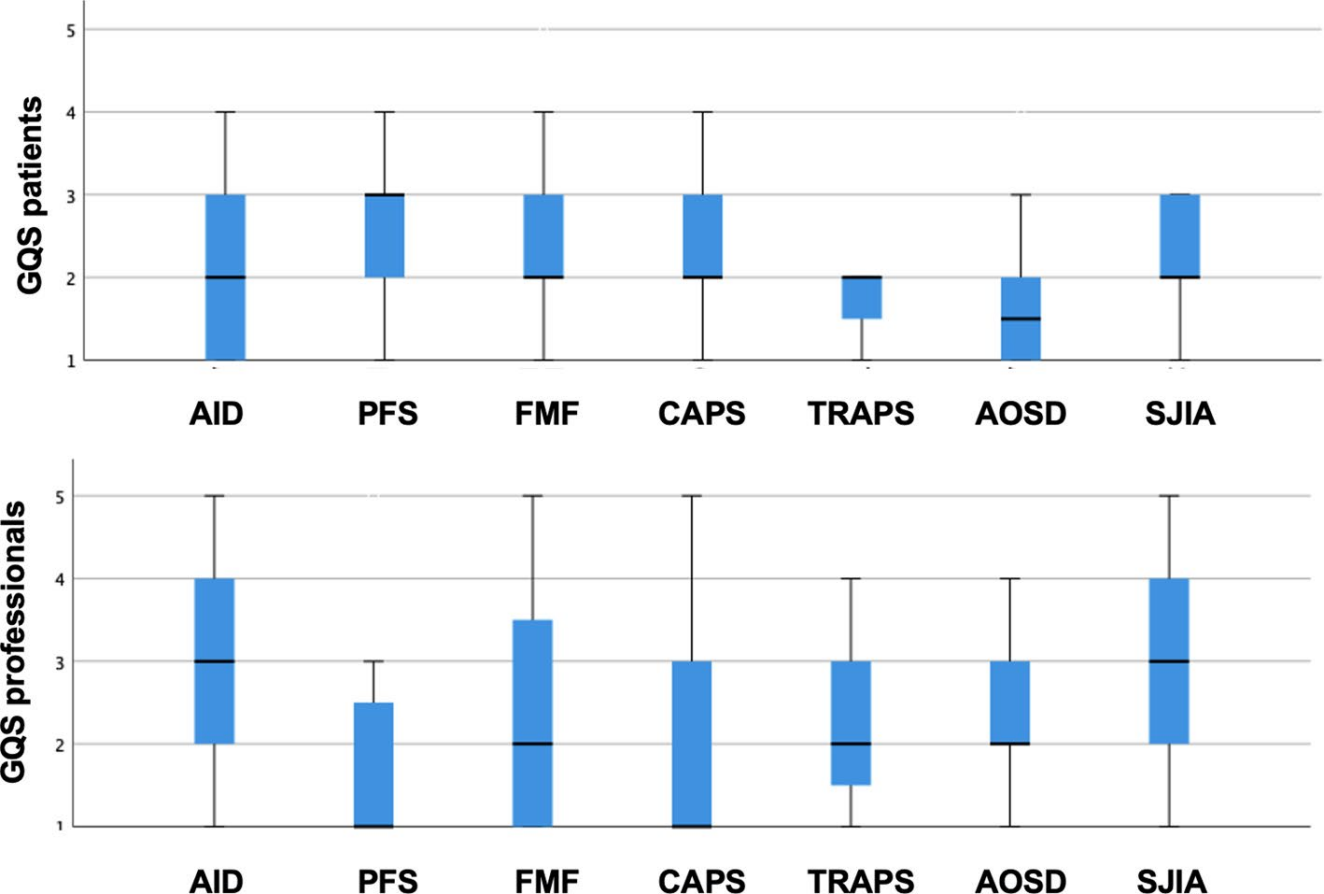
Boxplot showing the median and spread of the GQS for patients and professionals for subgroup analyses for *AID* autoinflammatory diseases, *PFS* periodic fever syndrome, *FMF* familial Mediterranean fever, *CAPS* Cryopyrin-associated periodic syndrome, *TRAPS* tumor necrosis factor receptor-associated periodic syndrome, *AOSD* adult onset of Still´s disease and *SJIA* systemic juvenile idiopathic arthritis

Subgroup analyses focusing on the individual search terms found that AID and SJIA videos achieved the highest scores (AID and SJIA: median 3), whereas CAPS and PFS videos showed the lowest scores (CAPS and PFS: median 1), all minimum 1 and maximum 5 for professionals (Fig. 3).

Videos created by patients and relatives were in general found to be of low quality for both target groups (87.5% for patients, 100% for professionals). Even videos created by health care professionals often showed low quality for patients (64.2%) as well as for professionals (43.2%).

Video duration was significantly longer in videos targeting a professional audience (*p* < 0.001) with a medium duration of 14.4 min (0.58–86.3 min) versus videos targeting patients with a median duration of 3.4 min (0.6–54.5 min). Concerning the GQS for professionals, length of the video significantly correlated with higher quality (*r* = 0.38, *p* < 0.001).

Focusing on the difference between the GQS for professionals dependent on whether the person who uploaded the video was a private person or a professional, there was a trend, but the difference did not reach statistical significancy. The median of the GQS was higher when uploaded by professionals than by private persons and relatives (median GQS for professionals uploaded by professionals = 3 (min 1, max 5); median GQS for professionals uploaded by a private person = 1 (min 1, max 2); (*p* = 0.072), while there was no significance for the GQS for patients.

The comparison between the GQS for patients and the GQS for professionals did not reach significancy (*p* > 0.1).

### Reliability of the videos

**Fig. 4:**
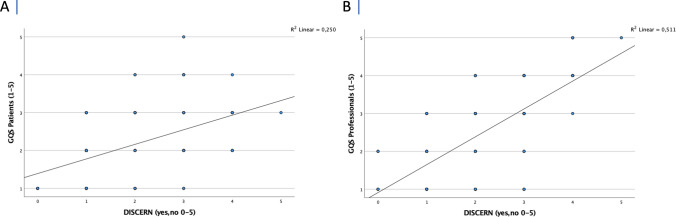
Correlation analyses of **A** GQS patients (1–5) with DISCERN score and **B** GQS professionals (1–5) with DISCERN score

A positive correlation was seen between video quality and reliability. The DISCERN score correlated significantly with the GQS for patients (*r* = 0.5, *p* < 0.001) as well as with the GQS for professionals (*r* = 0.72, *p* < 0.001). Videos with a high quality showed also a good reliability. Correlation analyses are shown in Fig. 4.

### The video power index

The median video power index (VPI) for all videos was 1.43 (min 0, max 48.78). The highest median was found for videos on FMF (median 2, min 0.46, max 10.46), whereas the lowest median was seen in videos on CAPS (median 0.72, min 0, max 16.11) and TRAPS (median 0.82, min 0.66, max 1.22). The group differences did not achieve significancy (*p* > 0.05) (see Figure SI 2 in the Supplementary Information).

## Discussion

This study was conducted to investigate whether patients or physicians can potentially benefit from the use of social media platforms like YouTube as a source of information on AID. Our analysis found that two-thirds of the videos on AID were uploaded to inform healthcare professionals on the diseases and only one-third targeted patients or their relatives. This is probably due to the fact that only a small proportion of uploaded videos (7.6%) was produced by patients or relatives themselves. The majority of the uploaded videos (78.1%) were produced by (and for) healthcare professionals.

The results of our analysis on AIDs differ from those of recently published YouTube analyses on other rheumatic diseases like gout or systemic sclerosis where the target audience were mostly patients [12, 15]. This might be due to a proportionally smaller number of patients suffering from AID in relation to other rheumatic diseases and the lack of specific patient organizations. Singh et al. [13] evaluated YouTube videos concerning rheumatoid arthritis and found that most of the videos were uploaded by private persons (36.3%). Zengin et al. [14] evaluated YouTube videos concerning biologic therapy and found similar results that most videos were uploaded by private persons (35.1%). Unlike these previous studies, videos on AID were very rarely uploaded by patients (7.6%). This could be caused by a limited access to information for AID patients in general, which conversely suggests that a lack of information leads to uncertainties in the first place and might therefore make it less attractive for patients or relatives to share information.

We also performed a subgroup analysis to capture both the patient’s and the healthcare professional´s part individually: For instance, videos dealing with the biochemical pathways in AID might be useful for viewers with a medical background, but are difficult to relate to without medical knowledge. Based on the GQS, most of the videos for patients showed a low quality (64.8%), and notably, little information was identified without preconditioned medical knowledge that might be relatable to patients. Interestingly, the quality of videos for professionals showed a similar pattern; here, 54.3% were of low quality. Nevertheless, 22.9% of the videos for professionals were of high quality, while only 7.6% of the videos showed a high quality for patients. As most of the videos were produced by medical professionals, this is an important finding highlighting the fact that there seems to be too little expertise and general knowledge on the topic among this group. Furthermore, it emphasizes that YouTube currently fails to provide sufficient high-quality content on AID that patients with no medical background could benefit from.

Across all videos, there was a significant negative correlation between the length of the video and the number of clicks of the video (*r* = − 0.196, *p* < 0.05). This could be related to the fact that longer videos are less attractive for users in view of time limitations or the assumption that the longer a video is, the more complicated it is.

However, video duration was significantly longer in videos targeting a professional audience with a medium duration of 14.4 min versus videos targeting patients with a median duration of 3.4 min (*p* < 0.001). Concerning the GQS for professionals, length of the video was significantly correlated with higher quality (*r* = 0.38, *p* < 0.001). In accordance with that result, previous studies found that videos with high quality had longer video duration [14, 15, 18]. This finding may be related to the fact that longer videos are more detailed and comprehensive than short ones [14]. In addition, this could be an indicator that longer videos are more suitable for professionals to convey quality information. This may also reflect the complexity of rare diseases and the variety of clinical manifestations.

Subgroup analyses focusing on the individual search items found that videos with the search terms ‘autoinflammatory diseases’ and ‘systemic juvenile idiopathic arthritis’ achieved better quality videos for professionals than the other search items. This suggests a more general interest and availability of quality information on these more common rare diseases. In line with previous studies [14], we found a positive correlation between the DISCERN score and the GQS for patients (*r* = 0.5, *p* < 0.001) and also for professionals (*r* = 0.72, *p* < 0.001), indicating that videos with high quality are at the same time more reliable and accurate.

Videos uploaded by professionals and targeting professionals had a higher GQS compared to private persons or relatives (*p* = 0.072). This is also concordant with recent studies [12, 15, 18] and suggests that the source of the videos is important for the quality and reliability of the content [15]. Therefore, medical societies and experts in the field should focus on this aspect in professional educational initiatives.

For videos uploaded by professionals in comparison to those by private persons, there were smaller numbers of likes/dislikes which indicates less engagement with professional content.

In today's world, social media play an increasingly important role as an information portal [19] not for patients’ education but also for networking and interaction between medical professionals [8]. YouTube in particular is now the most widely used video-sharing website with an average of more than 2 billion views per day [10]. Through its round-the-clock accessibility, it offers an easy and convenient way of obtaining information in comparison to a face-to-face encounter with a physician. In addition, it offers automatized subtitles as a setting and is so even more attractive for populations with language barriers.

Since autoinflammatory symptoms occur in childhood and adolescence due to innate genetic mutations, there is a particular risk of unfavorable outcomes for adolescent patient groups with AID [20]. Because of the early onset of the disease symptoms, younger users in particular search the Internet for information about their disease [21]. This underlines the importance of adequate information on AID, especially given a higher digital literacy among younger users [10]. While seeking information, however, patients do not tend to check the uploading source on digital platforms; the viewership and reliability of videos on YouTube are not linked to each other [22]. Previous studies investigating the quality and reliability of videos available on YouTube in rheumatology found poor or wide variations in the quality of the videos [15, 23–26]. Zengin et al. [14] found that videos about the side effects of biological therapy in rheumatic diseases had 40.3% high quality and 36.4% low quality. This result shows that the video platform has the potential to provide videos of good quality. However, predominantly investigations on more common rheumatic diseases were assessed, i.e., gout or osteoarthritis with comparably well-established expertise in rheumatology. To the best of our knowledge, there have been no investigations with regard to autoinflammatory diseases so far. In addition, private persons may consider videos less helpful if they require a medical background for understanding, whereas physicians may not benefit from the content and vice versa. Therefore, we conducted subgroup analyses for these two groups.

## Limitations

In line with previous studies, we conducted our investigation at a fixed time setting. YouTube is a platform with dynamic changes in content, since new videos are constantly uploaded. Although we used default settings for our search, the order of relevance for each search term might differ depending on the individual user history and location. Since consumers tend to watch only the first few pages of a search term [7], we limited our evaluation to the first 20 videos of each search term. In addition, only English language videos were analyzed and no other social media video platforms were searched.

## Conclusion

In 2021, YouTube announced to establish panels on authorized health content in cooperation with health organizations to increase the accessibility of credible, high-quality health-related content on their platform [27]. Unfortunately, our data underline that YouTube currently mostly fails to use its potential as an educational source on AID that both patients and professionals could benefit from. In the future, videos should be produced that are scientifically correct in terms of content and easy to understand for patients and their relatives. For these rare disease groups in particular, educational videos on YouTube could contribute to empower both patients and physicians and therefore help to facilitate their journey.

## Supplementary Information

Below is the link to the electronic supplementary material.

Supplementary file1 (DOCX 152 KB)

Supplementary file2 (DOCX 206 KB)
