# Supplementary figures and images for: Digital health information on autoinflammatory diseases: a YouTube quality analysis
Source: Rheumatol Int. 2022 Nov 14;43(1):163–71. doi: 10.1007/s00296-022-05243-9 (PMC9839787; doi:10.1007/s00296-022-05243-9)

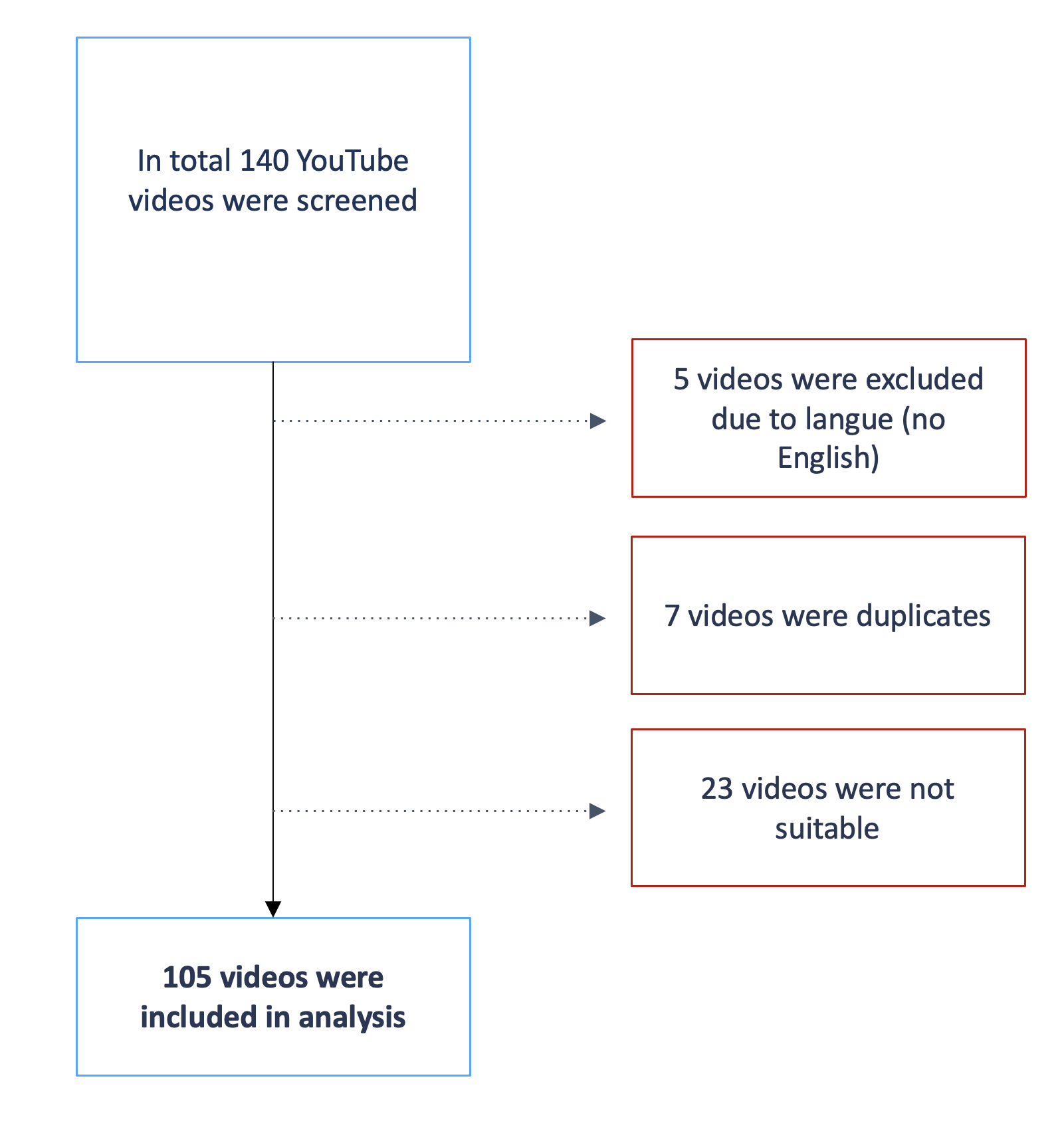


**Figure SI 1**: Flow chart of the selection process of YouTube videos

.

Supplement: Supplementary file 1 — Supplementary file1 (DOCX 152 KB) [file 296_2022_5243_MOESM1_ESM.docx]
