# Supplementary material for: Digital health information on autoinflammatory diseases: a YouTube quality analysis
Source: Rheumatol Int. 2022 Nov 14;43(1):163–71. doi: 10.1007/s00296-022-05243-9 (PMC9839787; doi:10.1007/s00296-022-05243-9)
Supplement: Supplementary file 2 — Supplementary file2 (DOCX 206 KB) [file 296_2022_5243_MOESM2_ESM.docx]

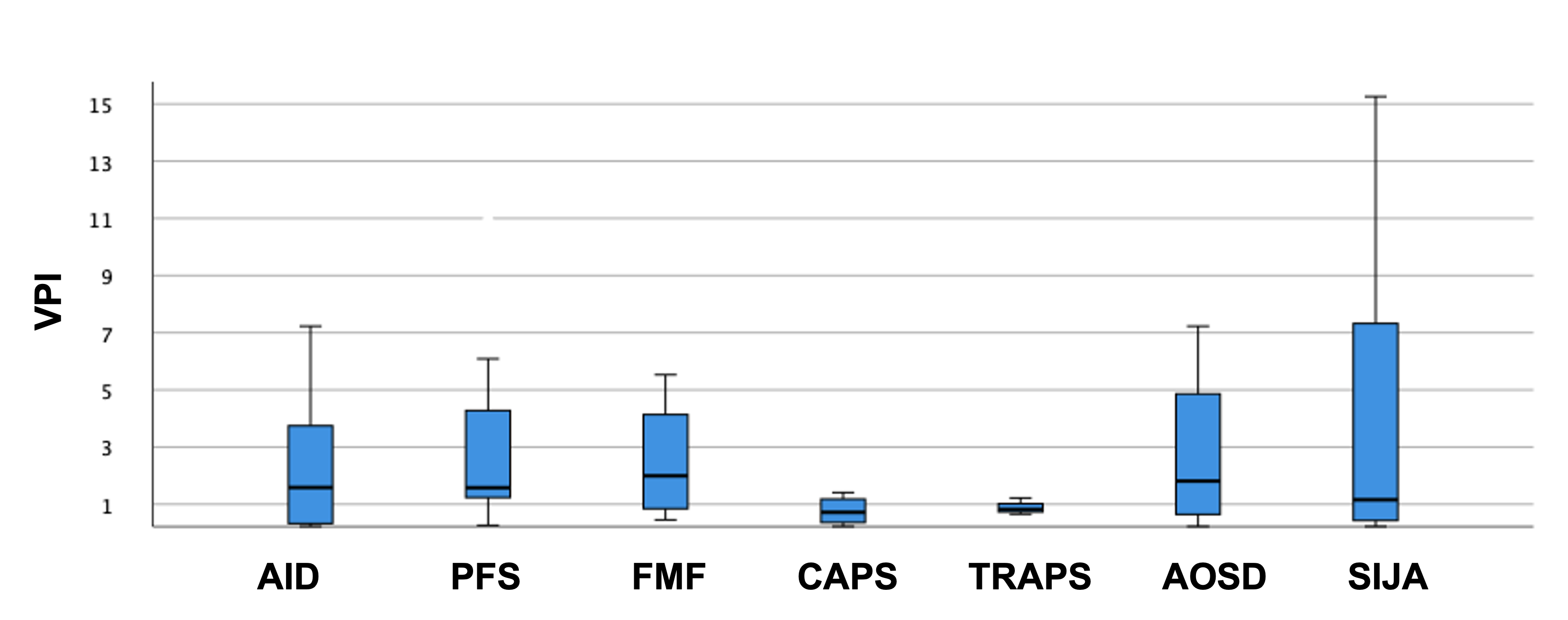


**Figure SI 2**: Boxplot showing the median and spread of the video power index (VPI). This graph does not present outliers and extreme values for a better overview.
